# Supplementary material for: Genes Located on 18q23 Are Epigenetic Markers and Have Prognostic Significance for Patients with Head and Neck Cancer
Source: Cancers (Basel). 2019 Mar 21;11(3):401. doi: 10.3390/cancers11030401 (PMC6468360; doi:10.3390/cancers11030401)
Supplement: Supplementary file 1 [file cancers-11-00401-s001.pdf]

Supplementary Materials: Genes Located on 18q23 Are Epigenetic Markers and Have Prognostic Significance for Patients with Head and Neck Cancer

Kiyoshi Misawa, Takeharu Kanazawa, Daiki Mochizuki, Atsushi Imai, Masato Mima, Satoshi Yamada, Kotaro Morita, Yuki Misawa, Kazuya Shinmura and Hiroyuki Mineta

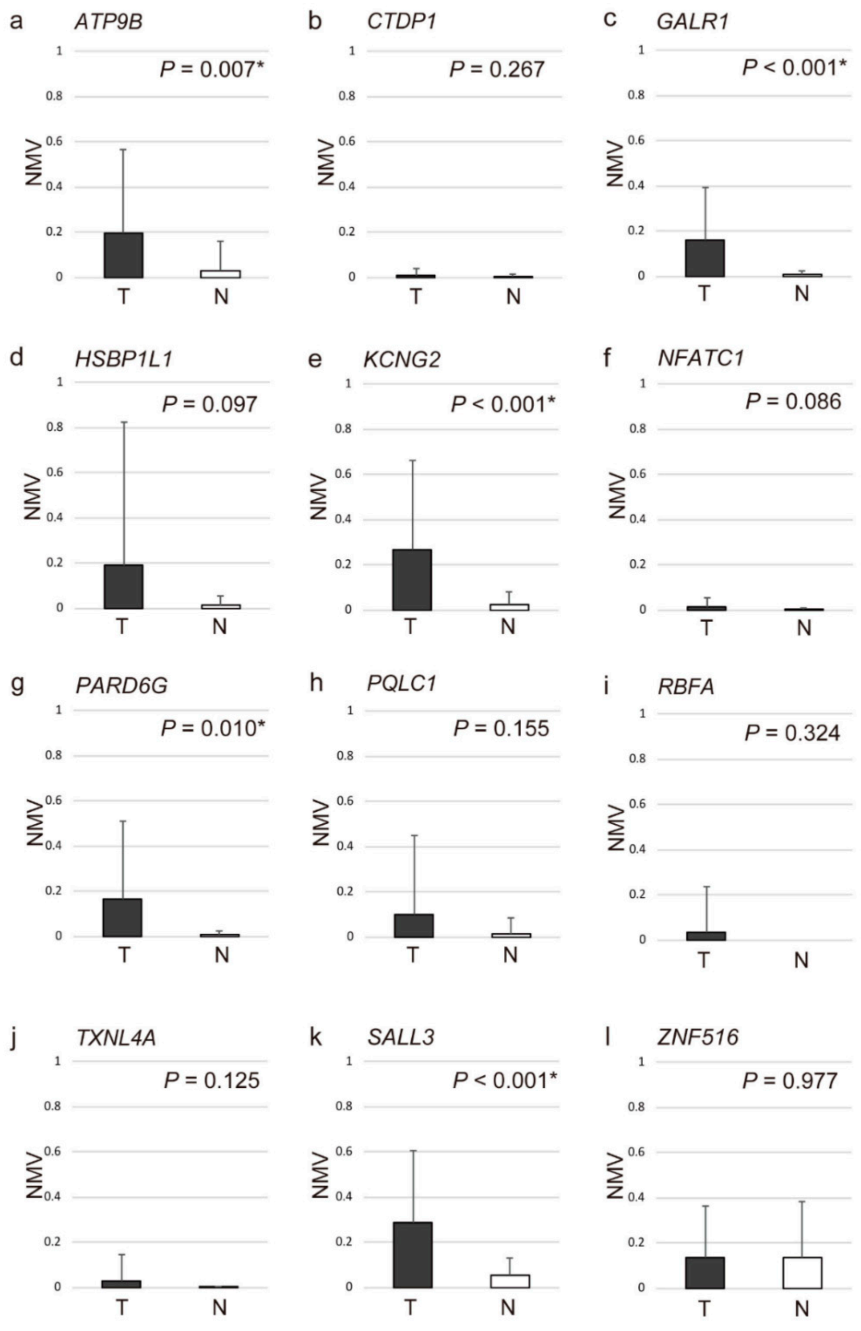

Figure S1. Methylation status in 36 matched pairs of head and neck tumor and adjacent normal tissue samples. The normalized methylation values (NMVs) for (**a**) ATP9B, (**b**) CTDP1, (**c**) GALR1, (**d**) HSBP1L1, (**e**) KCNG2, (**f**) NFATC1, (**g**) PARD6G, (**h**) PQLC1, (**i**) RBFA, (**j**) TXNL4A, (**k**) SALL3, and (**l**) ZNF516 promoters. T signifies head and neck tumor tissues. N indicates matched paired normal mucosal tissue.

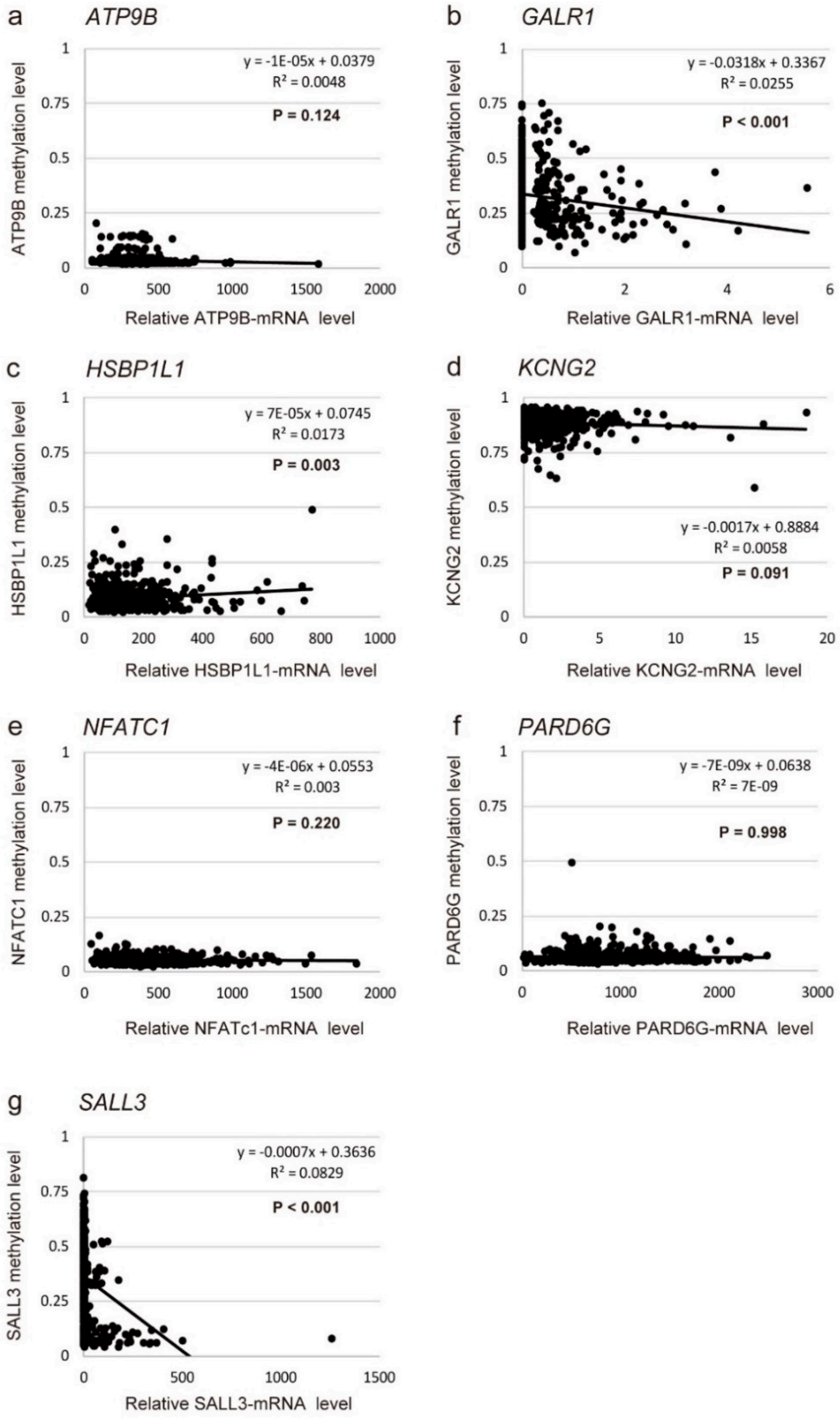

Figure S2. Methylation and expression status of the seven 18q23 genes in HNSCCs in the TCGA database. Scatter plot analysis of (**a**) *ATP9B*, (**b**) *GALR1*, (**c**) *HSBP1L1*, (**d**) *KCNG2*, (**e**) *NFATC1*, (**f**) *PARD6G*, and (**g**) *SALL3*. Spearman rank correlation coefficients (R2) and P values are shown.

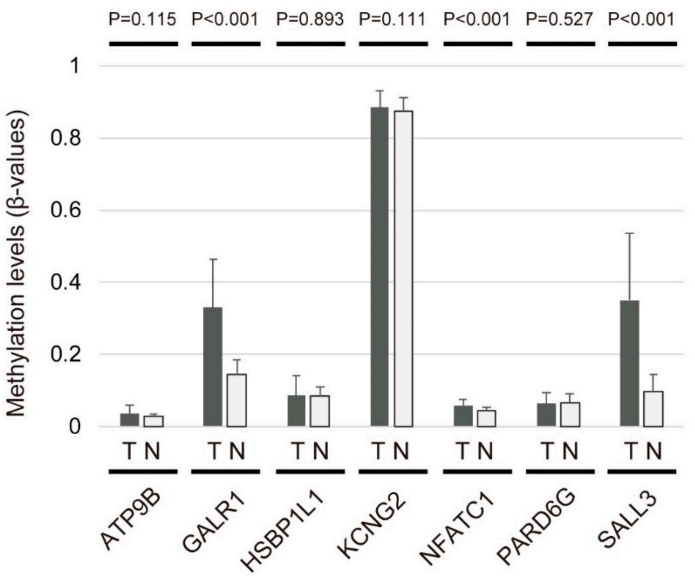

Figure S3. Methylation status of the seven 18q23 genes in HNSCC (T) and normal (N) samples in the TCGA database. DNA methylation data for *ATP9B*, *GALR1*, *HSBP1L1*, *KCNG2*, *NFATC1*, *PARD6G*, and *SALL3* were collected from the TCGA database.

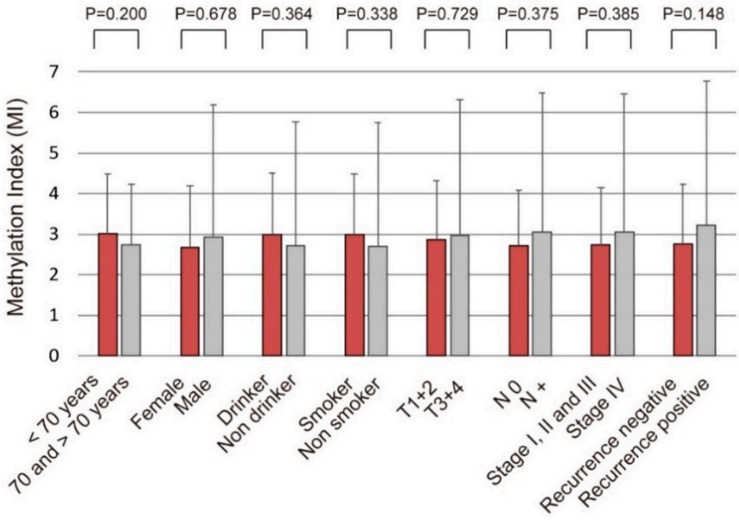

(**a**)

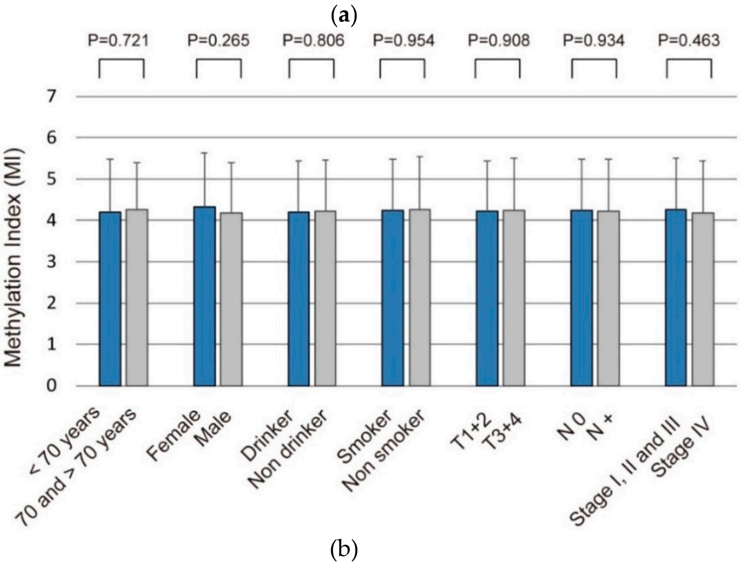

(b)

Figure 4. Association between methylation index (MI) and the selected clinical parameters. The mean Mis between the various groups was compared using a Student's t-test. Association between MI and

selected epidemiologic and clinical characteristics: (**a**) original cohort; no differences were noted with respect to any of the clinical characteristics; (**b**) TCGA cohort; there were no differences in clinical parameters. Mean and standard deviation are also indicated, and statistical comparisons between groups are depicted.

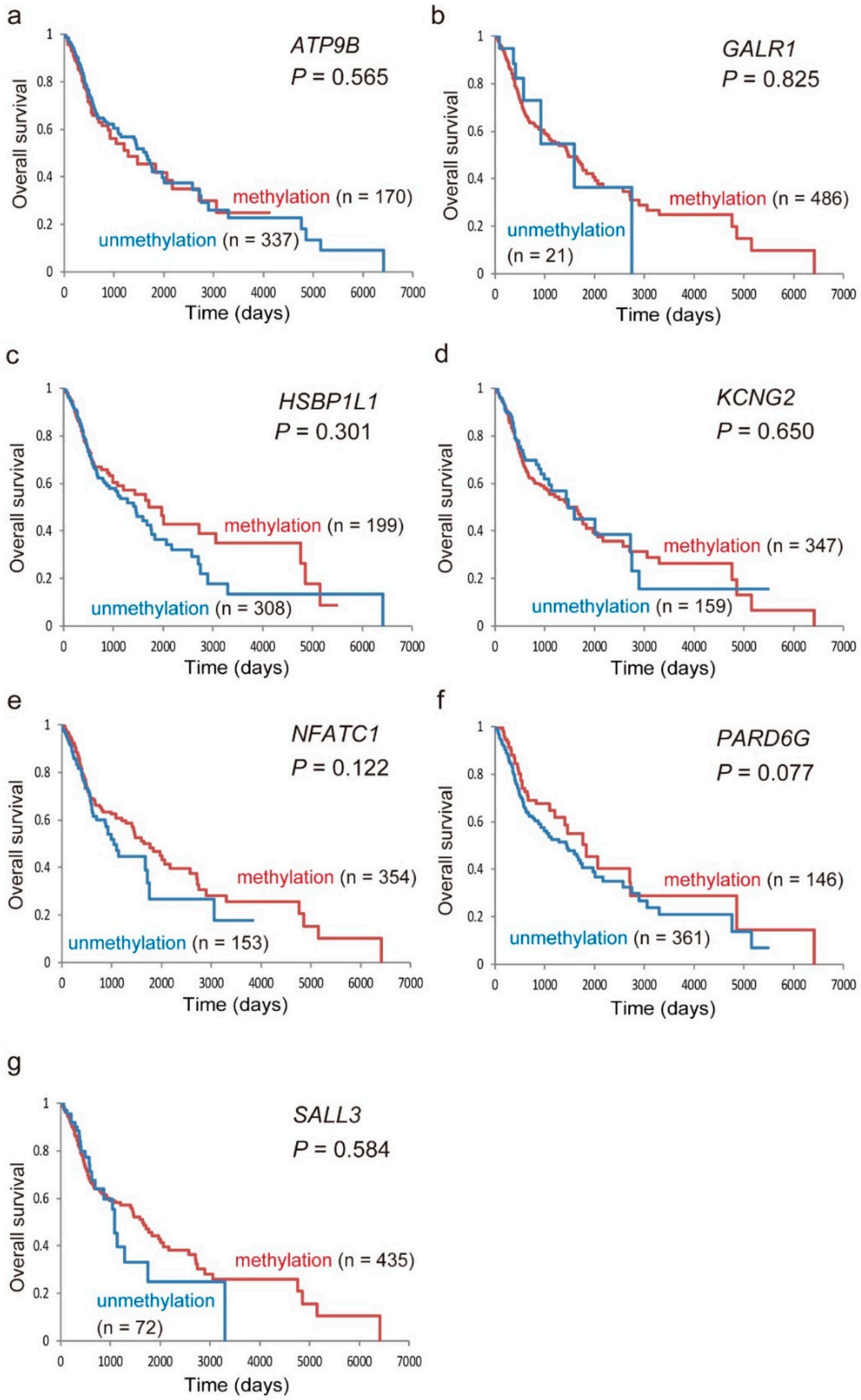

Figure S5. Kaplan–Meier survival curves based on TCGA data. Overall survival based on (**a**) ATP9B, (**b**) GALR1, (**c**) HSBP1L1, (**d**) KCNG2, (**e**) NFATC1, (**f**) PARD6G, and (**g**) SALL3 methylation; shown are cases of methylated (red lines) and unmethylated (blue lines) genes. A probability of < 0.05 (* $p < 0.05$) was considered a statistically significant difference.

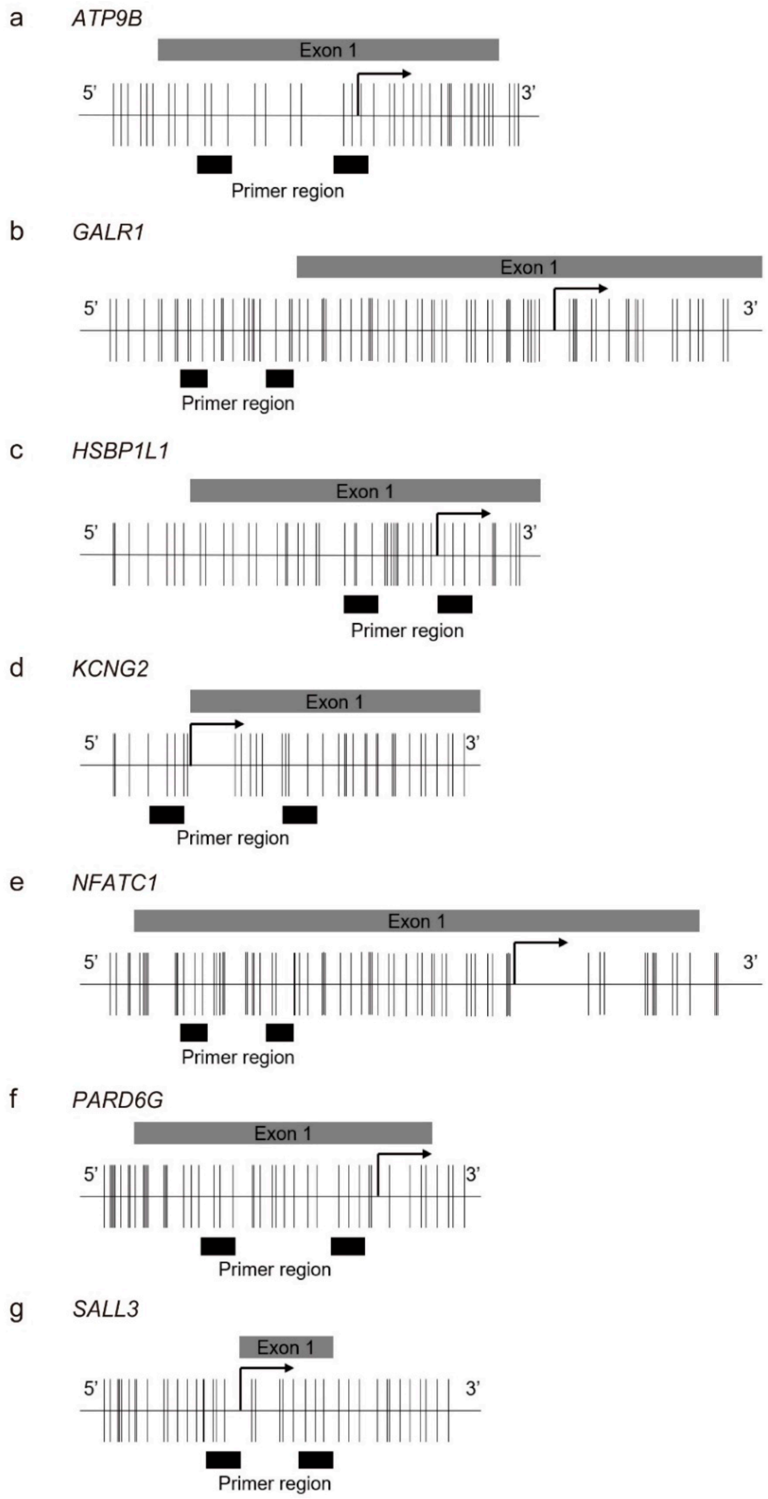

Figure S6. Schematic representation of (**a**) *ATP9B*, (**b**) *GALR1*, (**c**) *HSBP1L1*, (**d**) *KCNG2*, (**e**) *NFATC1*, (**f**) *PARD6G*, and (**g**) *SALL3* genes. CpG sites are within the expanded views of the promoter region. Vertical lines, individual CpG sites; black box, relative location of the primers used for Q-MSP; bent arrow, translation start site (ATG).

Table S1. Results of log-rank tests for effect of number of methylated genes on disease free survival in 243 HNSCC.

No. Methylated Genes	No. Patients with Profile	*p*
≥0	243	
≥1	231	0.318
≥2	201	0.095
≥3	141	0.143
≥4	92	0.583
≥5	34	0.056
≥6	10	0.098

Table 2. Real Time MSP Primer List.

Gene	Forward/Reverse	Sequence	Length (bp)
ATP9B	F	AGGCGGCGTTTTAGTCGGGGT	106
	R	CGCCATATTCCGACCGCCCCT	
CTDP1	F	TAGCGTAGGTTTCGTATCGAT	109
	R	CACCTCGACCACAACCGCCGT	
GALR1	F	GGTTCGCGGTATTCGGTAGT	99
	R	GGTTCGCGGTATTCGGTAGT	
HSBP1L1	F	GGAATCGCGGAGTTTAGTTGTCG	116
	R	CGAAAACTTCAAAACCCCGCACG	
KCNG2	F	AGGAGTTTTTCGGTTCGGTTCG	95
	R	TAATAATAACGTACCGAACGC	
NFATC1	F	CGAGATTTAGAGGTTTCGAATTCG	113
	R	ACCCTAAAACCTACGCGATAACTC	
PARD6G	F	GAGTAGTTCGTCGGTTAAGCG	114
	R	ATCAA CTCGCCGTCGCCCTC	
PQLC1	F	AGGTTTCGAGGTCGTAGGAGC	106
	R	TCCATCGCAACGCCCGCCTAA	
RBFA	F	ATACGTCGTTTGTTAGGCGTG	155
	R	CCCGCCGCAACCCACATAACG	
SALL3	F	GGGGTTCGAGCGTCGTTAGT	106
	R	CCGTACTCGAAAACCCCGTC	
TXNL4A	F	GTTAGTACGTACGGCGTTAG	113
	R	CCCGCACACGCAAACTCCGCT	
ZNF516	F	AGTTGTGTCGTTATTATCGGTCG	111
	R	TCGTAACCCCGACCGACCCTAAT	
ACTB	F	TGGTGATGGAGGAGGTTTAGTAAGT	133
	R	AACCAATAAAACCTACTCCTCCCTTAA	

Table 3. Results of the ROC curve analysis, the sensitivity, specificity, and cutoff value.

Genes	ROC Area	Sensitivity (%)	Specificity (%)	Cutoff Value
ATP9B	0.628	58.3	69.4	0.0049
GALR1	0.838	50	97.2	0.038
HSBP1L1	0.559	22.2	94.4	0.0562
KCNG2	0.718	75	69.4	0.0083
NFATC1	0.549	21.4	97.2	0.0178
PARD6G	0.636	30.6	97.2	0.0391
SALL3	0.786	58.3	91.7	0.11

© 2019 by the authors. Licensee MDPI, Basel, Switzerland. This article is an open access article distributed under the terms and conditions of the Creative Commons Attribution (CC BY) license (http://creativecommons.org/licenses/by/4.0/).
